# Drug-induced eosinophilic pneumonia: A review of 196 case reports

**DOI:** 10.1097/MD.0000000000009688

**Published:** 2018-01-26

**Authors:** Carmi Bartal, Iftach Sagy, Leonid Barski

**Affiliations:** Internal Medicine Division, Soroka University Medical Center, Ben-Gurion University of the Negev, Beer-Sheva, Israel.

**Keywords:** blood eosinophilia, drug induced, eosinophilic pneumonia, severity

## Abstract

**Background and objective:**

Eosinophilic pneumonia (EP) is an important subset of patients who present with pulmonary infiltrates and eosinophilia (PIE). EP is classified by chronicity and etiology and drug-induced EP is the main cause of secondary EP. The primary goal of this review was to examine all the case reports published since the syndrome was defined in 1990. It remains unclear whether acute or chronic EP (AEP or CEP) represent different diseases, and the secondary goal of this review is to determine if there are factors that may help distinguish these 2 entities.

**Methods:**

PubMed (MEDLINE and Medical Subject Headings) was searched for case reports of drug-induced EP or PIE syndrome published between 1990 and 2017. Case reports were only included if the diagnostic criteria for AEP or CEP were fulfilled. For each case, data were extracted pertaining to age, sex, type of medication associated with the disease, time from the onset of symptoms to diagnosis, eosinophil counts in the blood, eosinophil fractions in bronchoalveolar lavage (BAL) fluid, initial chest radiograph and computed tomography results, use of mechanical ventilation, and use of steroid treatment and recurrence.

**Results:**

We found 196 case reports describing drug-induced EP. The leading cause was daptomycin. From our review, we found that AEP is more common in younger patients with no gender preference. Eosinophilia in the blood at the time of diagnosis characterized only the CEP patients (80% in CEP vs. 20% in AEP). Abnormal findings on radiographic imagine was similar in both syndromes. A significant portion of AEP patients (20%) presented with acute respiratory failure requiring mechanical ventilation. Most patients with EP were treated with steroids with a higher rate of relapse observed in patients with CEP.

**Conclusion:**

AEP is a much more fulminant and severe disease than the gradual onset and slowly progressive nature of CEP. The pathogenesis of AEP and CEP remains unclear. However, there is significant clinical overlap among AEP and CEP that are associated with drug toxicity, suggesting the possibility that AEP and CEP are distinct clinical presentations that share a common pathogenic pathway.

## 1 Introduction

Eosinophilic pneumonia (EP), also known as pulmonary infiltrates with eosinophilia (PIE syndrome), is a rare and heterogeneous syndrome that is classified according to chronicity and etiology. This syndrome, which was first described in 1989, may be classified as acute EP (AEP) or chronic EP (CEP) and may be due to primary (idiopathic) or secondary causes.^[1]^ The currently used classification system of EP was described by Allen and Davis in 1994.^[2]^ There is some overlap in the characteristics of these 2 syndromes, including eosinophilic infiltration of the pulmonary parenchyma and clinical symptoms such as dry cough, dyspnea, chest pain, and fever. AEP is characterized by symptoms lasting less than 1 month and usually less than 1 week.^[3]^ Imaging findings typically include bilateral reticular ground-glass opacities that expand as the disease progresses. Patients with AEP generally present with neutrophilic leukocytosis without an elevated eosinophilic fraction. Differential cell counts of bronchoalveolar lavage (BAL) fluid shows >25% of all white blood cells in BAL fluid are eosinophils in both syndromes. Lung biopsy is rarely necessary to make the diagnosis of EP. In contrast to AEP, CEP has a gradual onset and the average time from onset to diagnosis is 5 months. The clinical features of CEP include cough (42%), which is usually productive, fever (67%), and dyspnea (80%) as well as B symptoms such as weight loss (60%) and night sweats (47%). Peripheral blood eosinophilia is commonly present and the absolute eosinophil count is usually greater than 1000 cells/μL. Additionally, total immunoglobulin E (IgE) levels are increased in 50% of the patients.^[4]^ Laboratory results commonly indicate a chronic inflammatory process with thrombocytosis and an elevated C-reactive protein level or sedimentation rate. Although there is some overlap, the imaging presentation of AEP is somewhat different and includes bilateral peripheral or pleural-based nonsegmental consolidative opacities and with less prevalence, ground glass opacities. These opacities are located in the upper lung zones in 50% of patients and are migratory in 25% of patients. Reticulation and nodules are not typical in cases of AEP, and the BAL fluid eosinophil fraction is almost always >25% and is >40% in 80% of cases.^[5]^

The main difference between AEP and CEP is that AEP usually has a fulminant presentation with severe hypoxemia. In some studies, more than 50% of patients with AEP required mechanical ventilation.^[4]^

The main causes of secondary EP include drugs and toxins. Less common causes include parasitic or fungal infection, transpulmonary passage of helminths (usually *Ascaris* species) and Loeffler syndrome. In this review, we analyze case reports of drug-induced EP published since 1990 to examine the hypothesis that AEP and CEP are different clinical entities.

## 2 Material and methods

Ethics approval was not sought for this retrospective review of previously published case reports.

PubMed (MEDLINE and Medical Subject Headings) was searched for all case reports describing drug-induced EP or drug-induced PIE syndrome published between 1990 and 2017. We excluded cases of toxin-induced EP, case reports for which the full text could not be acquired and duplicate cases. For each case, we collected data regarding age, sex, type of medication associated with the disease, time from onset of symptoms to diagnosis, eosinophil counts in the blood, eosinophil fraction in BAL fluid, initial chest radiograph and computed tomography results, use of mechanical ventilation, and use of steroid treatment and recurrence. Case reports were only included if the diagnostic criteria for AEP or CEP were fulfilled.

The diagnostic criteria for EP include respiratory complaints (dyspnea/cough/hypoxemia), pulmonary parenchymal infiltrates, peripheral blood eosinophilia, a BAL fluid eosinophil fraction >25% or histopathological results from a transbronchial biopsy and a negative work-up for other causes of peripheral blood eosinophilia.

AEP was defined by the presence of symptoms for <1 month at the time of diagnosis. If the symptoms began > 1 month before the time of diagnosis, we defined the syndrome as CEP. EP cases were defined as severe if patients had an oxygen saturation (SpO_2_) < 88% on room air and a respiratory rate >30 breaths/min, were admitted to the intensive care unit and required invasive or noninvasive mechanical ventilation. Moderate cases of EP were defined by dyspnea, SpO_2_ between 88% and 92% on room air and a respiratory rate between 20 and 30 breaths/min. Mild cases of EP were defined by dyspnea, SpO_2_ >92% on room air and a respiratory rate <20 breaths/min. For statistical comparisons, we divided all of the case reports into 2 groups (AEP and CEP).

### 2.1 Statistical analysis

Statistical analysis was conducted using SPSS version 23.0 (IBM Corporation, Armonk, NY). We investigated demographic, clinical, imaging, and therapeutic differences between cases of AEP and CEP. Descriptive statistics (frequencies, means, and standard deviations) were used to characterize the study sample. The χ^2^ statistics and independent samples *t* test were used to compare categorical and continuous variables between patients with AEP and CEP. Statistical significance was set at *P* values < .05.

## 3 Results

Drug-induced EP is a rare condition. In total, we found only 228 cases reported between 1990 and March of 2017. Ultimately, 196 full-text case reports met the criteria to be included in our analysis.

### 3.1 Associated drugs

Many medications were implicated in drug-induced EP (Table 1) and the most commonly cited drugs were daptomycin, mesalamine, sulfasalazine, and minocycline.

**Table 1:**
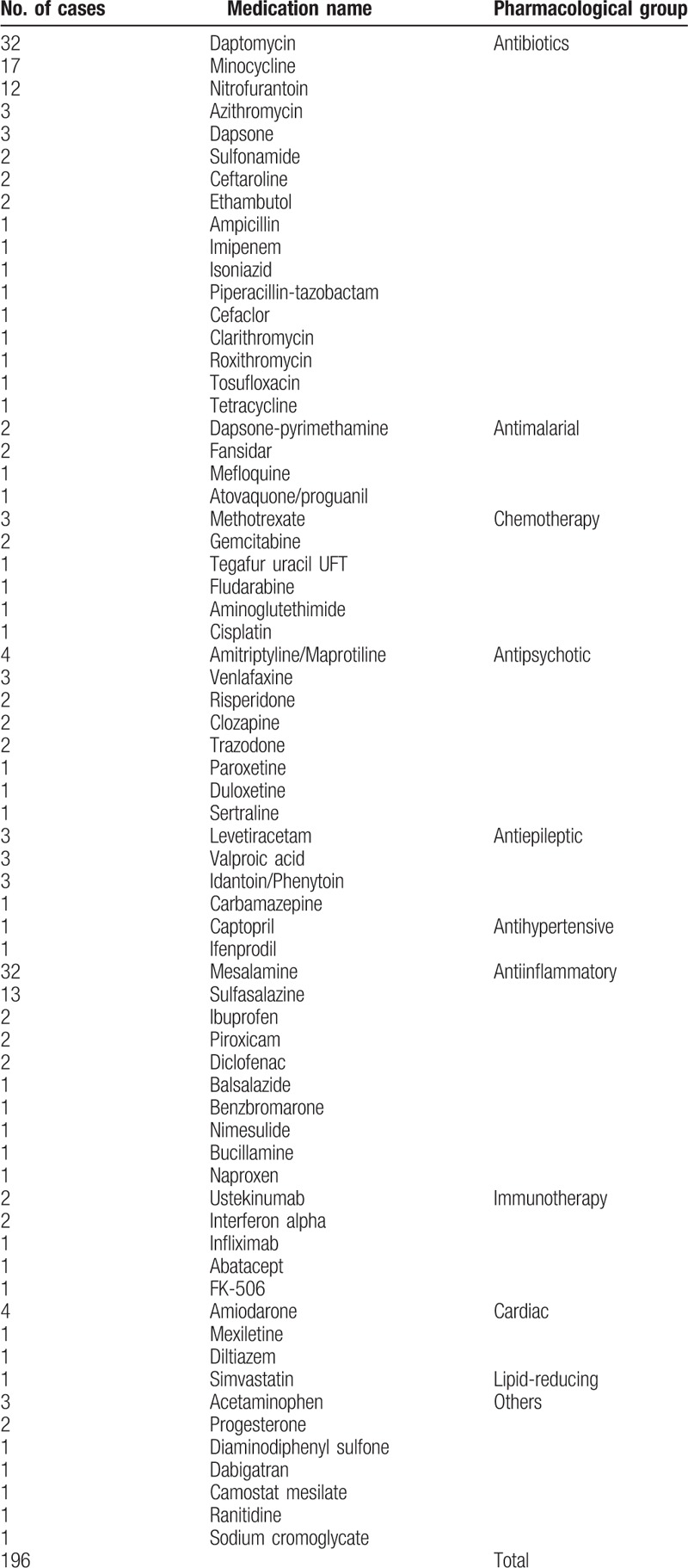
Drugs associated with eosinophilic pneumonia.

### 3.2 Clinical and laboratory findings

Table 2 shows a comparison of clinical and laboratory findings between the 2 groups. AEP was more commonly reported in the medical literature than CEP. The prevalence of syndromes was not significantly different between the sexes. The mean and median ages were significantly lower in the AEP group compared with the CEP group (48 and 38 years for the AEP group versus 56 and 44 years for the CEP group). The average time from the onset of symptoms to diagnosis was 9.8 days for the AEP group and 4.14 months for the CEP group. Peripheral blood eosinophilia was common in both types of EP (69% of cases with AEP and 77.6% of cases with CEP). There was no significant difference in the peripheral blood eosinophil count between the AEP and CEP groups (1232 cells/μL in the AEP group and 1490 cells/μL in the CEP group).

**Table 2:**
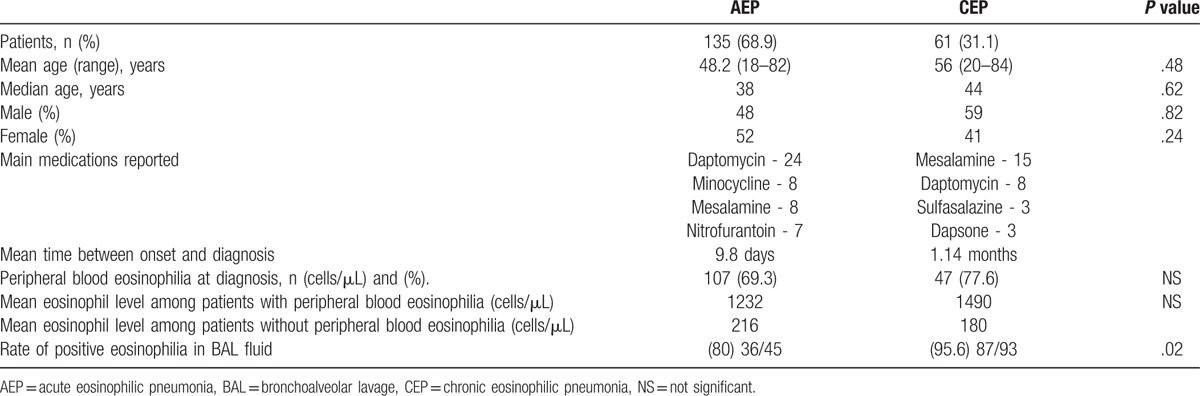
Demographic, clinical, and laboratory results of the acute eosinophilic pneumonia and chronic eosinophilic pneumonia groups.

BAL was performed in only 70% of the cases in this study. Significantly more patients with CEP who underwent BAL had an eosinophil fraction >25% in the BAL fluid compared with the AEP group (95% vs 80%; *P* = .02).

### 3.3 Imaging

Table 3 compares the imaging findings between the AEP and CEP groups. The 2 groups shared some imaging features in common. In the AEP group, 30% of cases had bilateral peripheral infiltrates with segmental consolidation. The second most common imaging finding in the AEP group was diffuse reticular or interstitial findings (26.7%), which were detected in only 14.7% of patients with CEP. Diffuse ground-glass infiltrates were detected in 11% of patients with AEP but in none of the patients with CEP. In CEP, the main imaging finding was bilateral peripheral segmental infiltrates, observed in 49% of patients. Wandering peripheral consolidations were seen in 10% of patients with CEP but in none of the patients with AEP. A few patients in each group presented with pleural effusion in addition to parenchymal findings (2 cases of AEP and 3 cases of CEP).

**Table 3:**
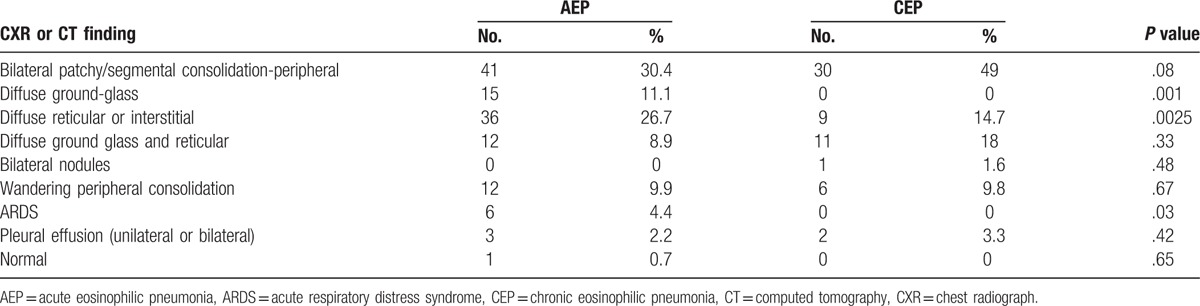
Comparison of imaging findings between patients with acute and chronic eosinophilic pneumonia.

### 3.4 Severity of the clinical presentation

A comparison of the severity of the clinical presentation between the 2 groups is summarized in Table 4. Among the patients with CEP, 96.7% presented with mild severity and mild respiratory distress. Conversely, 20.8% of AEP patients presented with severe respiratory distress and 19.2% required mechanical ventilation. Six patients with AEP who required mechanical ventilation presented with acute respiratory distress syndrome (ARDS), which was diagnosed radiographically. From 1994 to 2012, the diagnosis of ARDS was based on the American–European Consensus Conference criteria^[6]^ and the Berlin definition of ARDS has been used since 2012.^[7]^

**Table 4:**
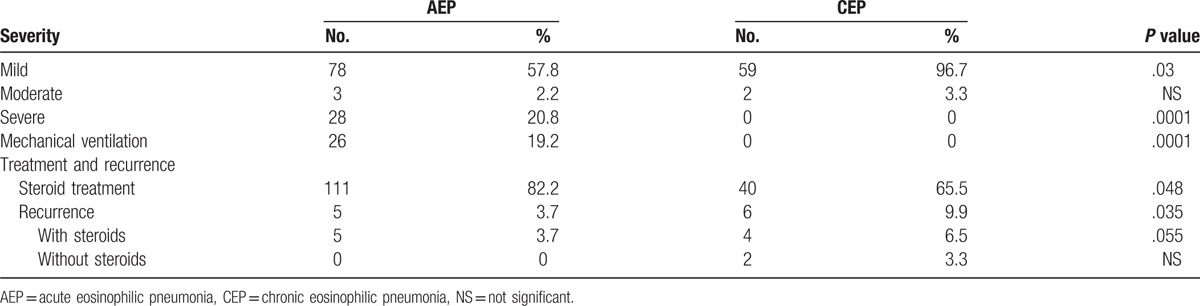
Comparison of severity, use of steroid treatment, and recurrence.

As expected, significantly more patients were treated with steroids in the AEP group compared with the CEP group (82.2% vs 65.5%; *P* = .015). In the 34.5% of CEP patients who were not treated with steroids, treatment consisted of cessation of the implicated medication. Furthermore, the recurrence rate was significantly higher in patients who were treated with steroids than in those not treated with steroids (6.5% vs 3.7%; *P* = .027). Recurrence has been defined by the authors as recurrent symptoms and the need for re-treatment at any time after stopping initial treatment.

## 4 Discussion

Although EP is a rare disease, we found that almost every family of medication was implicated. Moreover, we found a higher prevalence among a few pharmacological families such as antibiotics,^[8,9]^ nonsteroidal anti-inflammatory drugs^[10,11]^ and antiepileptic drugs,^[12]^ which suggests the possibility that there is a connection between the pathogenesis of EP and the mechanism of action of those commonly used medications.^[10,13]^

### 4.1 Pathogenesis

The cause of AEP and CEP remains unknown. It is also unclear whether these 2 entities share common pathophysiological mechanisms. One theory suggests that AEP is a hypersensitivity reaction to an unidentified inhaled antigen.^[14]^ This theory has been strengthened by several reports regarding inhaled cigarette smoke,^[15]^ airborne sand in military personnel deployed in Iraq^[16]^ and other environmental factors.

While the triggering factor of AEP is unknown, this syndrome is currently believed to be the result of eosinophilic-specific chemoattractants, including eotaxin, T-cell-expressed chemokines, and interleukin-5 released from T2 lymphocytes. The role of serotonin in the pathophysiology of asthma is well established, as 5-HT2A stimulates different signaling pathways and regulates cytokine release in airway epithelial cells.^[17]^ 5-HT2A has also been reported to play a role as a mediator of inflammation in the immune response outside the central nervous system.^[18]^ Both serotonin and eotaxin have been recently described as having an eosinophil chemoattractant profile, which may explain the connection between the pathogenesis of AEP and CEP and antipsychotic and antiepileptic drugs.^[12]^

Other mechanisms have also been suggested for drug-induced EP. Certain drugs, especially nitrofurantoin^[19]^ and daptomycin,^[11]^ have been reported to cause oxidant injury. Direct cytotoxic effects on alveolar capillary endothelial cells, injury mediated by deposition of phospholipids within the cell and immune-mediated injury causing symptoms of systemic lupus erythematosus have also been reported.^[20]^ Mesalamine, one of the leading causes of drug-induced EP, is believed to cause damage through immune-mediated alveolitis, as evidenced by lymphocytic infiltration.^[21]^

Moreover, amiodarone toxicity of the lung has been extensively investigated. Amiodarone toxicity may present as CEP, and some of the mechanisms of injury could explain how other drugs induce EP. Amiodarone toxicity is suggested to result from a combination of different mechanisms, such as (i) a “cytotoxic” effect to type-II pneumocytes as well as other cells of the lung parenchyma, such as inflammatory cells, endothelial cells, and fibroblasts; (ii) an “immune”-mediated mechanism in genetically predisposed patients and (iii) activation of the angiotensin enzyme system.^[22,23]^

These toxic mechanisms lead to a disruption of the lysosomal membranes by amiodarone molecules through protein C activation and the subsequent release of toxic oxygen radicals; oxygen radicals may induce activation of the caspase pathways and lead to apoptosis of lung epithelial cells. Furthermore, over the past decade, in vitro studies in both primary cultures of rat type-II pneumocytes and the human A549 alveolar epithelial cell line have shown that amiodarone induces alveolar epithelial cell apoptosis that is abrogated by antagonists of angiotensin II.^[24–27]^

Both the “cytotoxic” and the “immune” pathophysiological mechanisms could either independently or in combination lead to different forms of lung injury. In this review, we were unable to elucidate the main pathophysiological mechanism of EP syndromes; however, the evidence supports a multifactorial etiology.

### 4.2 Demographic features

A few intriguing clinical and laboratory results were observed in our investigation.

AEP has been reported to occur twice as often in men than in women^[28,29]^ and in CEP the ratio between men and women is reported to be equal.^[30]^ However, we did not observe these trends in our review. On the contrary, there was a trend toward male predominance among the patients with CEP and cases of AEP were equally distributed between men and women. As previously reported, AEP patients tend to be younger (20–40 years old),^[28,29]^ which is consistent with our findings.

### 4.3 Clinical features

AEP is a severe and dramatic syndrome. This explains the early detection (average of 9.8 days after the onset of symptoms). In comparison, the mean time to diagnosis of CEP is 4.14 months. It has been reported that most patients with AEP are diagnosed within the first week, but the longer time to diagnosis of CEP emphasizes the difficulty in diagnosing this syndrome in an outpatient setting. The severity of AEP is evidenced by the fact that 21% of patients had acute hypoxemic respiratory failure and 19.8% required mechanical ventilation. None of the patients with CEP had respiratory failure and 97% had only mild respiratory symptoms. Our results are consistent with those of Philit et al,^[31]^ who reported that 63% of patients had respiratory failure.

### 4.4 Imaging findings

After analyzing the radiographic findings, there was some degree of overlap between the 2 syndromes; however, we were unable to find a common pattern. Peripheral segmental/patchy bilateral infiltrates were more commonly seen in patients with CEP than in those with AEP (49% vs 30.4%), and this result was close to statistical significance (*P* = .06). These infiltrates have been previously described as being prototypical of CEP.^[32]^ In addition to peripheral segmental/patchy bilateral infiltrates, reticular/interstitial findings have been described significantly more often in cases of AEP than in cases of CEP (26.7% vs 14.7%; *P* <.025). Pleural effusion with parenchymal lesions was rarely found in either group (2.2% of AEP cases and 3.3% of CEP cases). Diffuse ground-glass opacities and an ARDS presentation were exclusively found in the AEP group (11% and 4.4%, respectively). Wandering infiltrates in both lungs is a unique finding of EP and was present in similar rates in both groups.

### 4.5 Eosinophil counts

Our data showed that peripheral blood eosinophilia was common in the initial presentation of CEP (77% of patients) with an average absolute eosinophil count of 1490/μL. In contrast, patients with AEP generally presented with an initial neutrophilic leukocytosis with no elevated fraction of eosinophils. As the disease progresses, the eosinophil count increases.^[3,33]^ In this study, only 20.7% of patients with AEP had eosinophil counts >500 cells/μL and the average among those with initial peripheral blood eosinophilia was 1390 cells/μL.

The diagnostic criterion of an eosinophil fraction >25% in the BAL fluid was observed in both syndromes, but significantly more in AEP group (95.6% of cases in the AEP group and 80% of cases in the CEP group). No case report could define EP without the presence of either peripheral blood eosinophilia or an elevated eosinophil fraction in the BAL fluid.

### 4.6 Treatment and recurrence

There are no guidelines for treating these EP syndromes with steroids. The cure rate for both AEP and CEP was very high: 96.6% in the AEP group and 90.1% in the CEP group. In this study, the recurrence rate was significantly higher in the CEP group, which is similar to what has been previously described.^[34,35]^ In the AEP group, recurrence was rare and the rate was lower than that anticipated based on previous descriptions of a small series of cases.^[36]^ Upon analyzing the use of steroid treatment, we found that 35% of CEP patients were treated only with cessation of the instigating drug, and the associated recurrence rate was only 3.3%. The patients with CEP who were treated with steroids (65%) had a higher recurrence rate (6.5%) and this difference was statistically significant. In the AEP group, the recurrence rate was much lower (3.7%) and most of the patients were treated with steroids (82%). There was no recurrence among AEP patients who were not treated with steroids. One possible explanation for this finding is that the patients with AEP who were not treated with steroids had a milder disease course.

Upon comparing patients treated with steroids in both groups, there was a significantly higher recurrence rate in the CEP group. It is logical to assume that the severity of the initial clinical presentation led to earlier steroid treatment, but the higher recurrence rate among patients with CEP treated with steroids may be due to premature discontinuation of steroid treatment. The optimal duration of steroid treatment for EP is unknown, but the more common approach is to slowly taper steroids over 2 to 3 months.^[33,34]^ Although steroid treatment resulted in a very high cure rate and a low recurrence rate in CEP cases, our data analysis revealed that cessation of the implicated drug resulted in no further need for steroid treatment. Steroid-sparing treatment with omalizumab may also be used in patients with recurrent disease if IgE levels are elevated; omalizumab has been recommended as a second-line treatment in patients with recurrence who did not improve with steroid treatment.^[37]^ Because most case reports did not report data regarding IgE levels and because the correlation between IgE levels and eosinophil counts has not been examined in a large series, we cannot currently support the use of treatments based on IgE levels.

### 4.7 Limitations

Due to the rarity of this syndrome, very few cases reports have been published during the past few decades, thus restricting the statistical power of this analysis. In addition, the heterogeneity of the published case reports decreases the quality of this data analysis.

## 5 Conclusions

In conclusion, there are more commonalities than differences between AEP and CEP. There is no doubt that AEP is a much more severe disease than CEP. Nevertheless, similarities in the blood eosinophil counts and the eosinophil fractions in BAL fluid, radiographic manifestations, and response rates to steroid treatment raises the possibility that both syndromes are on the same clinical spectrum. It is quite possible that, although they are generated by different drugs, both AEP and CEP share a common pathogenic pathway. Therefore, defining EP as AEP or CEP may have no practical use and only the severity of the EP presentation would determine the treatment. Most patients with a severe presentation of EP required mechanical ventilation, therefore, patients who are suspected of having severe EP should be monitored in an intensive care unit. On the basis of our findings, we recommend using a course of steroids with slow tapering over 2 to 3 months for all patients with EP and steroid treatment for 9 to 12 months for recurrent cases.

Regardless of age or sex, drug-induced EP should be suspected in any patient who has recently started taking a new medication and presents with new-onset dyspnea, bilateral infiltrates on chest radiograph, and peripheral blood eosinophilia with a negative eosinophilia work-up.

## Acknowledgments

The authors have no sources of funding to declare. We would like to thank Editage (www.editage.com) for English language editing.
